# Supplementary material for: Identification of predictive patient characteristics for assessing the probability of COVID-19 in-hospital mortality
Source: PLOS Digit Health. 2024 Apr 23;3(4):e0000327. doi: 10.1371/journal.pdig.0000327 (PMC11037536; doi:10.1371/journal.pdig.0000327)
Supplement: S1 Appendix — Table A. Summary of differences in the occurrence of delirium among the three age groups of patients. Table B. Statistical summary of the regression model shown in S1 Appendix. Equation G. Table C. Average marginal effect (AME), p-values, and confidence intervals associated with the factors incorporated in the benchmark model introduced in S1 Appendix. Equation G. The AME represents the average effect of a unit change in a predictor variable on the predicted outcome, averaged across all observations in the dataset. It provides a straightforward measure of the influence of each independent variable, revealing the expected average change in the dependent variable with a one-unit increase in that independent variable, while keeping all other variables constant. (PDF) [file pdig.0000327.s001.pdf]

Appendix

Identification of predictive patient characteristics for assessing the probability of COVID-19 in-hospital mortality

Bartek Rajwa[1,2*], Md Mobasshir Arshed Naved[3], Mohammad Adibuzzaman[4], Ananth Y. Grama[3], Babar A. Khan[5], M. Murat Dundar[6], Jean-Christophe Rochet[2,7*]

[1] *Bindley Bioscience Center, Purdue University, West Lafayette, IN, USA*
[2] *Purdue Institute for Integrative Neuroscience, Purdue University, West Lafayette, IN, USA*
[3] *Dept. of Computer Science, Purdue University, West Lafayette, IN, USA*
[4] *Oregon Clinical and Translational Research Institute, Oregon Health and Science University, Portland, OR, USA*
[5] *Regenstrief Institute, Indianapolis, IN, USA*
[6] *Dept. of Computer and Information Science, IUPUI, Indianapolis, IN, USA*
[7] *Borch Dept. of Medicinal Chemistry and Molecular Pharmacology, Purdue University, West Lafayette, IN, USA*

[*]Corresponding authors: brajwa@purdue.edu, jrochet@purdue.edu

https://doi.org/10.1371/journal.pdig.0000327

1 Elastic-net regularized logistic regression

The elastic net model has been represented as follows. Given a dataset with n observations $\{(x_i, y_i)\}_{i=1}^n$, where x_i is the feature vector for the i-th observation and y_i is the binary response variable (0 or 1), the elastic-net logistic regression model seeks to find the coefficient vector β:

$$\hat{\beta} = \underset{\beta\,\in\mathbb{R}}{\operatorname{argmin}} \left(-\frac{1}{n} \sum_{i=1}^{n} \left[y_i \log(\sigma(x_i^T \beta)) + (1 - y_i) \log(1 - \sigma(x_i^T \beta)) \right] + \lambda \left(\alpha \|\beta\|_1 + \frac{1-\alpha}{2} \|\beta\|_2^2 \right) \right) \tag{A}$$

where $\sigma(z) = \frac{1}{1+e^{-z}}$ is the logistic function, $\|\beta\|_1 = \sum |\beta_j|$ is the ℓ_1 norm of β, which is the sum of the absolute values of the coefficients.$\|\beta\|_2^2 = \sum \beta_j^2$ is the ℓ_2 norm squared of β, which is the sum of the squares of the coefficients, and λ is the tuning hyperparameter controlling the overall strength of the LASSO (ℓ_1) and ridge (ℓ_2) penalties, and α controls the balance between them [1].

Because the elastic net regularization penalizes the size of the coefficients, sets some irrelevant values to 0, and minimizes the impact of irrelevant features, the feature importance can be expressed straight from the model by the absolute values of the non-zero coefficients of the covariates.

It is important to recognize that minor adjustments in the random initialization or train-test split of the model may to considerable variances in the selected feature set for the majority of embedded feature selection methods. This problem is known as a selection instability [2,3]. In general, ℓ_1 regularization is known to be unstable [4]. However, investigating ensemble feature selection, in which the set of optimal features is produced from a collection of multiply independently trained models, helps resolve this issue. There are multiple approaches to increase the feature selection stability by performing various implementations of the ensemble approach, most notably the RENT model, which combines information about the frequency of feature occurrence and feature weights [2]. We followed a simple, yet effective, approach of training 10

independent models, each of which was initiated with a different random seed. To account for the data imbalance, we used the ROSE (Random OverSampling Examples) algorithm [5]. The simulated instances for training were generated *de novo* for each independent model.

2 XGBoost model

For a dataset $D = \{(x_i, y_i) : i = 1...n, x \in \mathbb{R}^m, y \in \mathbb{R}\}$ with n samples and m features, the predicted value $\hat{y}_i$ of the XGBoost model can be represented as:

$$\hat{y}_i = \sum_{k=1}^{K} f_k(x_i)\,,\, f_k \in F \tag{B}$$

where f_k represents a CART tree and the score given by the k-th tree to i-th data sample is denoted by $f_k(x_i)$. The set of K such functions is learned by minimizing the following objective function:

$$Obj = \sum_{l=1}^{n} l(y_i, \hat{y}_i) + \sum_{k=1}^{K} \Omega(f_k)\,,\text{where }\; \Omega(f) = \gamma T + \frac{1}{2}\lambda||w||^2 \tag{C}$$

Here, l is a convex training loss function that measures the difference between prediction $\hat{y}_i$ and target y_i; Ω is a model complexity function term that penalizes the complexity of the XGBoost model, where γ and λ are degrees of regularization. T and w refer to the number of leaves and the scores on each leaf of the tree, respectively. The XGBoost model can be trained in an additive manner. Given $\hat{y}_i{}^{(t)}$ as the prediction of the ith instance at t^{th} iteration, function f_t needs to be added to minimize the objective function:

$$Obj^{(t)} = \sum_{i=1}^{n} l(y_i, \hat{y}_i{}^{(t-1)} + f_t(x_i)) + \Omega(f_t) \tag{D}$$

By applying Taylor expansion this function is simplified as:

$$Obj^{(t)} = \sum_{i=1}^{n} [g_i f_t(x_i) + \frac{1}{2} h_i f_t(x_i)^2] + \gamma T + \frac{1}{2}\lambda \sum_{j=1}^{T} w_j^2 \tag{E}$$

where g_i and h_i are the first and second derivatives obtained on the loss function, respectively. By calling the stated tree creation model repeatedly, a large number of regression tree structures are acquired. The objective function, Obj, is then used to choose the optimal tree structure and insert it into the existing model to create the optimal XGBoost result.

3 Shapley values

Briefly, the exact Shapley values are computed based on the following procedure [6–8]. Let's consider an M-player cooperative game in which the objective is to maximize the payoff, and let $\mathcal{S} \subseteq M = \{1, ..., M\}$ be a subset of $|\mathcal{S}|$ players. Further, let's assume we have a contribution function $v(\mathcal{S})$ that maps subsets of players to real numbers, which we refer to as the worth or contribution of coalition $\mathcal{S}$. The worth of coalition S describes the expected total sum of payoffs that the members of $\mathcal{S}$ can obtain through cooperation [9]. The Shapley value is one method for distributing the total gains to the players, assuming that they are all cooperating. It is a "fair" distribution (i.e., characterized by efficiency, symmetry, null player property, and linearity) and is expressed as:

$$\varphi_j(v) = \varphi_j = \sum_{\mathcal{S} \subseteq \mathcal{M} \setminus \{j\}} \frac{|\mathcal{S}|!(M - |\mathcal{S}| - 1)!}{M!} (v(\mathcal{S} \cup \{j\}) - v(\mathcal{S})), \quad j = 1, \ldots, M \tag{F}$$

which is the weighted mean over contribution function differences for all subsets $\mathcal{S}$ of players not containing player j. Colloquially, the $\mathcal{S}$ values illustrate how important each player is to the overall cooperation and what payoff the player can reasonably expect from participation in the game. SHAP values provide a

straightforward way to determine which features contribute to a prediction by considering a model trained on a set of features as a value function on a coalition of players. Importantly, Shapley values may have causal interpretations where the conventional "conditioning by observation" as in Pearl's do-calculus, can be replaced by "conditioning by intervention" [10,11].

4 Model describing the probability of death.

$$\log\left[\frac{P(\text{Class}=\text{died})}{1-P(\text{Class}=\text{died})}\right] = \alpha + \beta_1(\text{Delirium}_{\text{Yes}}) + \beta_2(\text{Sex}_{\text{Female}}) + \beta_3(\text{Age}_{\text{Middle}}) + \beta_4(\text{Age}_{\text{Older}}) + \beta_5(\text{Race}_{\text{Black}}) + \beta_6(\text{Race}_{\text{Other/Unknown}}) + \beta_7(\text{Braden}) \tag{G}$$

5 Additional patient characteristics information and analysis

**Age-groups comparison**	**Sex**	**Odds ratio**	***p*-value**
[51.8, 66.9) vs. [17.1, 51.8)		1.52	0.679
[66.9,101.5] vs. [17.1, 51.8)	Male	2.11	0.285
[66.9,101.5] vs. [51.8, 66.9)		1.38	0.715
[51.8, 66.9) vs. [17.1, 51.8)		1.09	0.988
[66.9,101.5] vs. [17.1, 51.8)	Female	5.26	0.001
[66.9,101.5] vs. [51.8, 66.9)		4.82	0.004

Table A. Summary of differences in the occurrence of delirium among the three age groups of patients.

**Predictor**	
Class: died/survived	Log odds (SE)
Delirium symptoms	1.655^{**} (0.339)
Female	-0.707^{*} (0.319)
Age [51.8, 66.9)	2.486^{*} (1.058)
Age [over 66.9]	4.440^{**} (1.028)
Black	-0.158 (0.317)
Other	0.108 (0.785)
Braden score	-0.043^{*} (0.021)
Constant	-4.330^{**} (1.081)
Observations	471
Log Likelihood	-136.291
AIC	288.583
R^2 Tjur	0.282
Note:	$^{*}p<0.05$; $^{**}p<0.01$

Table B. Statistical summary of the regression model shown in Equation G

Factor	AME	p-value	LCL	UCL
Age [51.8, 66.9)	0.072	0.002	0.027	0.117
Age [over 66.9]	0.322	0.000	0.251	0.393
Braden score	-0.004	0.041	-0.008	0.000
Delirium symptoms	0.187	0.000	0.105	0.268
Black	-0.014	0.620	-0.071	0.043
Other	0.010	0.892	-0.139	0.159
Female	-0.063	0.022	-0.118	-0.009

Table C. Average marginal effect (AME), p-values, and confidence intervals associated with the factors incorporated in the benchmark model introduced in Equation G. The AME represents the average effect of a unit change in a predictor variable on the predicted outcome, averaged across all observations in the dataset. It provides a straightforward measure of the influence of each independent variable, revealing the expected average change in the dependent variable with a one-unit increase in that independent variable, while keeping all other variables constant.

References

[1] Zou H, Hastie T. Regularization and Variable Selection via the Elastic Net. Journal of the Royal Statistical Society: Series B (Statistical Methodology). 2005;67(2):301–320. doi:10.1111/j.1467-9868.2005.00503.x.

[2] Jenul A, Schrunner S, Liland KH, Indahl UG, Futsæther CM, Tomic O. RENT—Repeated Elastic Net Technique for Feature Selection. IEEE Access. 2021;9:152333–152346. doi:10.1109/ACCESS.2021.3126429.

[3] Nogueira S, Sechidis K, Brown G. On the Stability of Feature Selection Algorithms. Journal of Machine Learning Research. 2018;18(174):1–54.

[4] Xu H, Caramanis C, Mannor S. Sparse Algorithms Are Not Stable: A No-Free-Lunch Theorem. IEEE Transactions on Pattern Analysis and Machine Intelligence. 2012;34(1):187–193. doi:10.1109/TPAMI.2011.177.

[5] Menardi G, Torelli N. Training and Assessing Classification Rules with Imbalanced Data. Data Mining and Knowledge Discovery. 2014;28(1):92–122. doi:10.1007/s10618-012-0295-5.

[6] Shapley LS. A Value for N-Person Games. In: Roth AE, editor. The Shapley Value: Essays in Honor of Lloyd S. Shapley. Cambridge: Cambridge University Press; 1988. p. 31–40.

[7] Song E, Nelson BL, Staum J. Shapley Effects for Global Sensitivity Analysis: Theory and Computation. SIAM/ASA Journal on Uncertainty Quantification. 2016;4(1):1060–1083. doi:10.1137/15M1048070.

[8] Owen AB, Prieur C. On Shapley Value for Measuring Importance of Dependent Inputs. SIAM/ASA Journal on Uncertainty Quantification. 2017;5(1):986–1002. doi:10.1137/16M1097717.

[9] Aas K, Jullum M, Løland A. Explaining Individual Predictions When Features Are Dependent: More Accurate Approximations to Shapley Values. Artificial Intelligence. 2021;298:103502. doi:10.1016/j.artint.2021.103502.

[10] Pearl J. Causality: Models, Reasoning and Inference. 2nd ed. Cambridge, U.K. ; New York: Cambridge University Press; 2009.

[11] Janzing D, Minorics L, Bloebaum P. Feature Relevance Quantification in Explainable AI: A Causal Problem. In: Proceedings of the Twenty Third International Conference on Artificial Intelligence and Statistics. PMLR; 2020. p. 2907–2916.
