# Supplementary material for: Identification of predictive patient characteristics for assessing the probability of COVID-19 in-hospital mortality
Source: PLOS Digit Health. 2024 Apr 23;3(4):e0000327. doi: 10.1371/journal.pdig.0000327 (PMC11037536; doi:10.1371/journal.pdig.0000327)
Supplement: Attachment — Submitted filename: Response to the reviewers.pdf [file pdig.0000327.s002.pdf]

<u>Editor's comments</u>

We thank the editor for the encouraging evaluation and express our gratitude for the meticulous scrutiny of our manuscript. The editor's keen eye detected several errors that had evaded our careful proofreading efforts.

Equations would best appear only in an appendix. Technical terms should be defined/explained briefly.

We have relocated the mathematical descriptions of the methods to the Appendix section.

Section 2.5 contains a lot of ideas but is is too technical, difficult to follow and hard to relate to the problem at hand.

We have moved the technical details of SHAP analysis to the Appendix of the document. Additionally, we have included in the text a simplified interpretation of the concept that will allow readers to better understand the use of Shapley values in the context of clinical feature selection.

I appreciate the effort made in producing high quality figures. They could be better leveraged with enhanced captions and comments in the main text.

Thank you! We corrected and enhanced the captions.

p.6 table 5: Please define Average marginal mean and how it is computed (btw, isn't it Average Marginal Effect as suggested by the second column name?)

Yes, indeed. It is a mistake. The column header correctly specified the values as an average marginal effect. We corrected the Table caption.

There are two logistic regression (LR) models here (sections 2.3 and 3.1) and it is not always clear which model it is referred to later in the text. Also the wording GLM is sometime used instead of LR. Please choose one wording and stick to it consistently.

Indeed, we have a simple regression (non-regularized) model introduced in Section 3.1 and the elastic-net LR model, which is used for feature selection. We revised the text to ensure that these two models are not confused, and we mention the GLM framework only in the context of non-regularized models.

p.6 fig 1 the caption refers to \gamma while it should presumably be \lambda

Again, thank you for spotting the mistake! The caption was corrected.

p.7 what is the "LS algorithm"?

Thank you for bringing this error to our attention. We apologize for the confusion. We meant to refer to LR-ENET (elastic net regularized logistic regression), which was used for feature selection in our work. We have made the necessary revisions to the text.

Reviewer 1

We thank the reviewer for providing thoughtful comments that have enabled us to improve the manuscript. The points raised by the reviewer (and the resulting changes made to the manuscript) are outlined in detail below.

The part on "Dataset description" should be in Results section. The Methods section should include a description of (i) how the data was acquired (ii) what were the inclusion and exclusion criteria and (iii) what variables were collected, including a clear description of the outcome variables.

We moved the tables so their placement corresponds to the relevant sections.

Inclusion Criteria: Age ≥ 18 years, diagnosed with COVID-19, admitted to the ICU; Exclusion Criteria: None. The study was observational, encompassing all patients hospitalized between March 2020 and September 2020. Due to the de-identification process, exact admission dates are not available.

Data were extracted from electronic health records (EHR), encompassing a comprehensive array of patient status descriptors amounting to several hundreds of features. It is crucial to clarify that the variables analyzed in this study were not predetermined but emerged as a result of a rigorous feature filtering and selection process, described in detail in the “Methods” section. As mentioned in the manuscript, our analysis focused on descriptors associated with the initial evaluation conducted within the first 24 hours of patient admission.

Our approach to data analysis was fundamentally data-agnostic. We initially considered all quantifiable readings from the EHR. The primary step in our analysis involved the exclusion of variables with near-zero variance and those with substantial missing inputs. Subsequently, the remaining variables underwent a feature importance analysis to identify the most significant predictors. All the relevant extracted predictors are described in the “Results” section.

Patient characteristics are described in the results when compared between survivors and non-survivors. This is already a univariate analysis for testing association with mortality. However patient characteristics should be compared between the categories of the main independent variable, which is delirium. Such analysis is typically useful for indicating confounders and other effects to be accounted for in the multivariate analysis.

See the response to the following question in which we address the issue of delirium.

As to the findings regarding delirium – could they be just another representation of an older age? Although age was included in the multivariate analysis, were there cases at all among the younger patients? A possible answer could be obtained by an additional subgroup analysis, assessing the effect of delirium among the older patients group only.

Multiple reports have demonstrated that severe COVID-19 disproportionately affects elderly patients. Therefore, it is understandable that this group has a higher proportion of older patients. Indeed, according to our observations, delirium is more likely to occur among older female (but not male) patients; however, a further grouping within this cohort does not indicate that the emergence of delirium can be solely attributed to age.

Here is an additional table that groups the patients into three age categories, with 157 patients in each group. As the table demonstrates, cases of delirium were observed in each of the age groups. A version of this Table has been added to the revised manuscript as Table 3).

	Females			Males		
**Age group**	**No Delirium**	**Delirium**	**Total**	**No Delirium**	**Delirium**	**Total**
**[17.1, 51.8)**	92.3% (84)	7.7% (7)	91	89.4% (59)	10.6% (7)	66
**[51.8, 66.9)**	91.7% (66)	8.3% (6)	72	84.7% (72)	15.3% (13)	85
**[66.9,101.5]**	69.5% (57)	30.5% (25)	82	80.0% (60)	20.0% (15)	75
**Total**	**84.5% (207)**	**15.5% (38)**	**245**	**84.5% (191)**	**15.5% (35)**	**226**

Regarding the specific quantification of the delirium occurrence in different age groups of females and males, the Table (shown in Appendix as Table A1) below summarizes the analysis:

Age-group comparison	Sex	Odds ratio	p-value
[51.8, 66.9) vs. [17.1, 51.8)	Male	1.52	0.679
[66.9,101.5] vs. [17.1, 51.8)		2.11	0.285
[66.9,101.5] vs. [51.8, 66.9)		1.38	0.715
[51.8, 66.9) vs. [17.1, 51.8)	Female	1.09	0.988
[66.9,101.5] vs. [17.1, 51.8)		5.26	0.001
[66.9,101.5] vs. [51.8, 66.9)		4.82	0.004

Although there is a substantial difference between the occurrence of delirium in different age groups for females, the effect is less pronounced (and not statistically significant) for males.

The delirium cases are also spread between two major racial groups, as the table below demonstrates:

	White			Black		
**Age group**	**No Delirium**	**Delirium**	**Total**	**No Delirium**	**Delirium**	**Total**
**[17.1, 51.8)**	88.9% (64)	11.1% (8)	72	94.1% (64)	5.9% (4)	68
**[51.8, 66.9)**	90.0% (54)	10.0% (6)	60	86.7% (78)	13.3% (12)	90
**[66.9,101.5]**	71.9% (46)	28.1% (18)	64	76.1% (67)	23.9% (21)	88
**Total**	**83.7% (164)**	**16.3% (32)**	**196**	**85.0% (209)**	**15.0% (37)**	**246**

However, overall, there was no statistically significant difference between the occurrence of delirium in white and African American sub-cohorts (p=0.52)

Known risk factors for COVID-19 mortality were not accounted for. Were all patients comorbidity free?

We have addressed the issue of comorbidities in the added “Limitations” section. Briefly: It is widely recognized that COVID-19 has a greater impact on patients with preexisting health conditions [1–6]. The analysis of patient data from Indiana performed by the Regenstrief Institute reveals that hypertension, chronic pulmonary disease, congestive heart failure, diabetes, and renal diseases were among the most common comorbidities in the group from which our cohort was drawn. In fact, the findings suggest that admission to the ICU was highly unlikely for patients without any comorbidities. The EHRs provided us with the patient’s medical history in the form of ICD10 codes, dating back to 2 years prior to the admission. However, the vast diversity in comorbidities present in the analyzed cohort renders these data non-actionable from a machine-learning perspective. Additionally, our objective was not to link specific

comorbidities to patient survival, as this topic has already been extensively researched. Rather, we aimed to identify easily recognizable clinical features that may indicate a decreased likelihood of patient survival, regardless of their condition.

"Importantly, Shapley values may have causal interpretations where the conventional “conditioning by observation” as in Pearl’s do-calculus, can be replaced by “conditioning by intervention” – please explain further – which of the effects is hypothesized as causal and why is it suitable to such definition. Was the causal effect estimated? If so please specify it clearly in the Results Section.

Shapley values, a concept borrowed from cooperative game theory, are used in our study to determine the importance or contribution of each clinical feature in a predictive model. The Pearl’s Do-Calculus (which we do not employ or report on) is a formal system developed by Judea Pearl for reasoning about causation in statistical models. It allows for the analysis of causal effects by manipulating variables within a model. In Pearl's framework, "do(x)" represents an intervention where one actively sets the value of a variable X, as opposed to just observing it.

In the context of our study, “conditioning by observation" describes the traditional framework when we assess the probability of an outcome given the observation of certain variables. For instance, observing that a patient shows signs of delirium and then estimating the probability of subsequent patient’s death. On the other hand, "conditioning by intervention" occurs when one can actively intervene to set a variable to a specific value and then observe the outcomes. For instance, intervening to lower the probability of delirium, actively changing the treatment of specific patients with delirium, and observing the change in the effect.

What we are trying to communicate is that if the interpretation of the Shapley values in a causal framework is mathematically justified, we can consider our results as preliminary evidence for a possible causal link. In simpler terms, we suggest that instead of just seeing how the presence of a feature correlates with the outcome, the computed Shapley values suggest that actively changing a feature (through an intervention) might affect the outcome. We are also aware of the critique of this interpretation among some computer scientists [7].

Obviously, we did not pursue any interventions in the reported work, and the study was entirely observational. Therefore, we cannot assess the strength of the putative hypothesized causal effect. However, due to the statistical interpretation of the feature importance scores we used, one could use the results as a convenient representation of highly plausible hypotheses that can (and should!) be tested in a study where intervention is available. Only then could the effect size be truly estimated. Therefore, our choice of metric that drove the feature selection makes the selected features good candidates for intervention-based studies.

Reviewer 2

We would like to thank the reviewer for offering an insightful critique that highlighted the limitations of our study. The reviewer's thoughtful inquiries prompted us to add a new subsection titled “Limitations” into our manuscript. This section serves to emphasize the concerns raised by the reviewer, ensuring that our paper's conclusions are not extrapolated beyond their appropriate scope.

Information on features definitions, engineering/grouping, what is available for selection, the coding system, what dataset is used and what is available in it (e.g. HES in the UK) – affects interpretation as a specific condition may have multiple distinct coding methods, diminishing their significance when examined separately.

The hospitals used a health information system provided by Cerner. The systems are designed to support:

- International Classification of Diseases (ICD): Cerner's electronic health record (EHR) systems are equipped to handle ICD codes for diagnosis and inpatient procedures (ICD-10-CM for clinical diagnosis and ICD-10-PCS for inpatient procedural coding).
- Current Procedural Terminology (CPT) and Healthcare Common Procedure Coding System (HCPCS): For documenting and billing outpatient and professional medical services and procedures, Cerner's systems can accommodate CPT and HCPCS codes.

The data were collected during the clinical care of COVID-19 patients and stored in the medical records of the hospital. The Regenstrief Institute, which acts as the data warehouse for relevant hospitals' electronic records, performed an unstructured data extraction [8,9]. The extracted data were then curated into patient-level variables to facilitate analysis.

Given the relatively small sample size (N=471 with 72 cases), the XGB model may risk overfitting and limited generalisability.

The reviewer is correct. The small group size means that our confidence regarding the prediction is only moderate. However, as we emphasized in the manuscript, the goal of using a classifier (whether it was XGBoost or Elastic Net regression) was to guide feature selection. In other words, the classifiers are useful only insomuch as they provide us with a reproducible and explainable methodology for feature selection during the training process. In our context, the features are deemed important if they drive the classification. Additionally, please note that two classifiers were used precisely because the feature selection guided by only one of the classifiers might be biased due to the risk of overfitting. Finally, the selected features were critically reviewed on the basis of the current understanding of the mechanisms and symptoms of COVID-19.

In summary, even if the classifiers are imperfect, the feature selection they perform is useful if used as a guide for subsequent critical review of these features. The potential overfitting may indeed bring potentially irrelevant features, but we did not see any features being highlighted that were not independently observed in other clinical cohorts in other studies.

Defining COVID-19 mortality / clarifying if patients were admitted for COVID or other reasons, e.g. fatally-ingested toxic substances, but when admitted, found to have COVID?

The cohort comprises patients who were admitted to hospitals in Indianapolis, IN, during the early phase of the pandemic. During this phase, patients were discouraged from seeking non-critical help due to the rapidly developing pandemic situation. It is not possible to retrospectively establish to what extent the deaths occurred due to COVID directly or due to a dramatically exaggerated immune response that coincided with patients' frailty related to their other comorbidities. This would require a careful inquiry and analysis of every individual case, possibly involving pathologists. We rely on electronic health records and the designations provided by the hospital. These definitions indeed evolved during the pandemic and are often retroactively revised.

The issue of incidental hospitalization of patients “with” COVID-19 and its influence on the model would be much more significant for data collected recently. Indeed, patients who suffer from COVID-19 and those who simply carry the virus often asymptomatically and are hospitalized for another reason can be distinguished, as demonstrated by Fillmore et al. [10]. We did not perform such an analysis.

However, from a practical standpoint, the verification of the extent to which COVID-19 was a small factor, a large factor, or a direct cause of death is irrelevant. Our analysis demonstrated that patients admitted to the hospital with COVID-like symptoms and showing particular manifestations during the first 24 hours are much more likely to die. We do not believe that some of these manifestations are necessarily COVID-limited. In fact, the appearance of delirium would be seen as a critical predictor for any other severe infectious disease. The same is true of symptoms that may arise from kidney damage. If our work leads to elevating the attention to particular symptoms that may be associated with higher mortality, then the actual underlying cause of these symptoms is of low importance as far as interventions can be designed to alleviate the proximal causes. On the other hand, the emergence of a pathogen to which most of the population is immunologically naïve will always increase the risk posed by the presence of other comorbidities, frequently resulting in symptoms that cannot be entirely disentangled in regards to a causal connection.

Data collection time period – needed to account for potential variations in outcomes due to evolving treatment strategies and COVID variants.

While the exact admission dates of patients in the dataset are not accessible due to the deidentification process, it is important to note that all the data were collected between March 2020 and September 2020. Although the samples were not genotyped, we surmise that the most probable variants affecting the patients in the research cohort were the original L strain of COVID-19 or one of the early mutations, including the S, V, GR, GH, or GV strains. Based on the location and timeline, it is highly probable that most of the patients were infected with the GR-strain variant [11–13].

Handling of missing data
Why this benchmarking model/particular features?

The approach was essentially assumption-free. As we tried to convey in our manuscript, our goal was not to build a predictive classifier for COVID-19 severity (which would be invalidated mainly by the evolution of the virus and the emergence of vaccination) but rather to identify the critical, observable features that may lead to better patient stratification and risk recognition. The broader goal was to show that the methodology could be used to recompute the critical features, given the emergence of new data.

For the initial selection, we took all the available features that did not show missingness or near-zero variance. Further, among the multiple repeated measurements available, we selected the features describing the patient's status in the first 24 hours after admission, and we took the minimal and maximal values of the observations. From this point on, the feature selection was driven entirely by the classifiers constrained by the information about mortality. Consequently, the feature selection was not performed by the analysts but driven by the data.

As mentioned in the manuscript, the choice of models (elastic net, XGBoost) was dictated by their previously reported high performance in feature selection. The use of the Shapely factor was inspired by its statistical interpretation, which incorporates the notion of causality. Additionally, as documented in the manuscript, the Shapley factor has previously been employed in the clinical context.

Why is 20 predictors set as the limit for elastic-net?

We based our decision on practical factors, such as the efficiency of computations, particularly when utilizing the outcomes for the subsequent SHAP analysis. Our initial investigation did not reveal any consistent and reliable informative characteristics beyond that count. In fact, as illustrated in Figure 3, the significance of the remaining clinical readouts was consistently minimal beyond the eighth feature.

Is oversampling (ROSE) done before creating bootstrap samples? This can introduce bias and artificially inflate the AUC, as the OOB samples overlap with the training sample.

We understand that the reviewer is inquiring whether the entire dataset in our setting was oversampled using ROSE before any training took place. We would like to clarify that this was not the case. The ROSE algorithm was employed during training instead. A bootstrap sample of the original dataset is generated for each iteration. Subsequently, the ROSE algorithm is executed to generate synthetic samples for the minority class contained within that bootstrap sample. This step is conducted separately for each bootstrap iteration. Finally, the model is trained on the newly balanced dataset (post-ROSE application) and then evaluated.

We updated the text to clarify the confusing wording.

The manuscript's claims that predictive combinations of clinical features are causally linked to mortality or that the study produces easily understandable and actionable clinical features lack sufficient support based on the presented analysis. As the authors mentioned, the different approaches selected “very different sets of predictive features,” and that the selected features vary considerably depending on the seed. The selected features may also well be a proxy for other conditions.

Three important points must be emphasized in the response:

1) The generation of different subsets of predictive features when using sparse regularization methods is an expected and understandable result (See [14]). Indeed, we used multiple repeats (multiple seeds) for the classification training and feature selection to take advantage of the properties of the sparse selection methodology. The features reported were derived from averaging the results of multiple seeds. The figures depict the extent to which the features impacted the trained classifiers. Sparse methods have an inherent property of yielding varying orders of features or even slightly different features with different seeds. However, it is not a concern as informative feature sets may have multiple subsets that lead to the same classification success. Our features were quite stable, and we do not see it as an issue. If every rerun of the training/feature selection yielded the same result, it would indicate that most features are meaningless and do not contribute to the model's prediction.
2) The features are expected to be proxies or surrogate measures because most of them, by definition, are not mechanically tied to a single condition or comorbidity. Again, we do not see this as a problem, as general clinical features cannot, by their nature, be considered exclusive. For instance, an elevated temperature or high CRP level in the blood may point to multiple conditions that are manifested by a proximal cause, which is inflammation.
3) Specific clinical manifestations might be causally linked to mortality. However, we are discussing connections that are not exclusive. For instance, the emergence of kidney failure is associated with a higher level of mortality, and kidney failure can indeed be caused by multiple factors. However, multiple studies are now documenting the association between kidney failure and COVID-19 infection [15–20]. The EHR includes measures such as BUN that our feature selection process has identified. However, our feature selection procedure also identified the frequency of voiding as one of the critical predictive factors. Of course, we recognize that an abnormally high urination frequency is not “causing” mortality, and there is no direct causal link between COVID-19 infection and urination. Following the reviewer's critique, the voiding frequency in this context would be a proxy/surrogate measure related indirectly (although still causally) to the infection. However, the surrogate nature of this feature does not make it less important or useful. On the contrary, it makes this easily observable feature extremely valuable, as it can be evaluated in the hospital without any specialized equipment. The fact that the selection of features agrees with observations reported in the literature demonstrates not only the value of our approach for agnostic feature selection but also the practicality and applicability of the result.

Finally, it is essential to note that in isolation, none of the observations of the association between particular symptoms and poor outcomes is sufficient to demonstrate, without a doubt, the causal links. For

instance, in isolation, every one of the cited kidney injury reports would be insufficient. Only a carefully designed, sufficiently powered, randomized trial would provide us with a self-sustained body of evidence within a single study. However, our report, just like other reports documenting observational results, should be seen in the context of other supporting evidence. The fact that the EHR analysis demonstrates a link between acute kidney injury and COVID-related mortality increases the weight of the existing evidence and encourages the search for a mechanism. The current literature admits that the mechanism of COVID-related kidney injury still has not been fully elucidated. Yet, failing to aggregate evidence from multiple sources to update our beliefs or conclusions is a logical fallacy.

Following the reviewer's comment, we updated our discussion to emphasize that our observations should be considered in conjunction with other discussed papers that provide concurrently and independently obtained observations. We do not suggest that our models in isolation sufficiently provide links between the observed symptoms and outcomes

References

1. Guan W, Liang W, Zhao Y, Liang H, Chen Z, Li Y, Liu X, Chen R, et al. *Comorbidity and its impact on 1590 patients with COVID-19 in China: a nationwide analysis*. Eur Respir J. 2020;55(5). https://doi.org/10.1183/13993003.00547-2020
2. Williamson EJ, Walker AJ, Bhaskaran K, Bacon S, Bates C, Morton CE, Curtis HJ, Mehrkar A, et al. *Factors associated with COVID-19-related death using OpenSAFELY*. Nature. 2020;584(7821):430–6. https://doi.org/10.1038/s41586-020-2521-4
3. Sanyaolu A, Okorie C, Marinkovic A, Patidar R, Younis K, Desai P, Hosein Z, Padda I, et al. *Comorbidity and its impact on patients with COVID-19*. SN Compr Clin Med. 2020;2(8):1069–76. https://doi.org/10.1007/s42399-020-00363-4
4. Russell CD, Lone NI, Baillie JK. *Comorbidities, multimorbidity and COVID-19*. Nat Med. 2023;29(2):334–43. https://doi.org/10.1038/s41591-022-02156-9
5. Ng WH, Tipih T, Makoah NA, Vermeulen J-G, Goedhals D, Sempa JB, Burt FJ, Taylor A, et al. *Comorbidities in SARS-CoV-2 patients: a systematic review and meta-analysis*. mBio. 2021;12(1):10.1128/mbio.03647-20. https://doi.org/10.1128/mbio.03647-20
6. Booth A, Reed AB, Ponzo S, Yassaee A, Aral M, Plans D, Labrique A, Mohan D. *Population risk factors for severe disease and mortality in COVID-19: A global systematic review and meta-analysis*. PLOS ONE. 2021;16(3):e0247461. https://doi.org/10.1371/journal.pone.0247461
7. Ma S, Tourani R. *Predictive and causal implications of using Shapley value for model interpretation*. In: Proceedings of the 2020 KDD Workshop on Causal Discovery. PMLR; 2020. p. 23–38.
8. Dixon BE, Grannis SJ, McAndrews C, Broyles AA, Mikels-Carrasco W, Wiensch A, Williams JL, Tachinardi U, et al. *Leveraging data visualization and a statewide health information exchange to support COVID-19 surveillance and response: Application of public health informatics*. J Am Med Inform Assoc. 2021;28(7):1363–73. https://doi.org/10.1093/jamia/ocab004
9. *COVID-19 Dashboard*. Regenstrief Institute. https://www.regenstrief.org/covid-dashboard/
10. Fillmore NR, La J, Zheng C, Doron S, Do NV, Monach PA, Branch-Elliman W. *The COVID-19 hospitalization metric in the pre- and postvaccination eras as a measure of pandemic severity: A retrospective, nationwide cohort study*. Infect Control Hosp Epidemiol. 2022;43(12):1767–72. https://doi.org/10.1017/ice.2022.13
11. Hamed SM, Elkhatib WF, Khairalla AS, Noreddin AM. *Global dynamics of SARS-CoV-2 clades and their relation to COVID-19 epidemiology*. Sci Rep. 2021;11(1):8435. https://doi.org/10.1038/s41598-021-87713-x
12. van Dorp L, Acman M, Richard D, Shaw LP, Ford CE, Ormond L, Owen CJ, Pang J, et al. *Emergence of genomic diversity and recurrent mutations in SARS-CoV-2*. Infect Genet Evol. 2020;83:104351. https://doi.org/10.1016/j.meegid.2020.104351
13. Mercatelli D, Giorgi FM. *Geographic and Genomic Distribution of SARS-CoV-2 Mutations*. Front Microbiol. 2020;11. https://doi.org/10.3389/fmicb.2020.01800
14. Jenul A, Schrunner S, Liland KH, Indahl UG, Futsæther CM, Tomic O. *RENT—repeated elastic net technique for feature selection*. IEEE Access. 2021;9:152333–46. https://doi.org/10.1109/ACCESS.2021.3126429
15. Caceres PS, Savickas G, Murray SL, Umanath K, Uduman J, Yee J, Liao T-D, Bolin S, et al. *High SARS-CoV-2 Viral Load in Urine Sediment Correlates with Acute Kidney Injury and Poor COVID-19 Outcome*. J Am Soc Nephrol. 2021;32(10):2517. https://doi.org/10.1681/ASN.2021010059
16. Shao M, Li X, Liu F, Tian T, Luo J, Yang Y. *Acute kidney injury is associated with severe infection and fatality in patients with COVID-19: A systematic review and meta-analysis of 40 studies and 24,527 patients*. Pharmacol Res. 2020;161:105107. https://doi.org/10.1016/j.phrs.2020.105107
17. Solimando AG, Susca N, Borrelli P, Prete M, Lauletta G, Pappagallo F, Buono R, Inglese G, et al. *Short-Term Variations in Neutrophil-to-Lymphocyte and Urea-to-Creatinine Ratios Anticipate Intensive Care Unit Admission of COVID-19 Patients in the Emergency Department*. Front Med. 2021;7. https://doi.org/10.3389/fmed.2020.625176
18. Casas-Aparicio GA, León-Rodríguez I, Barrera CA la, González-Navarro M, Peralta-Prado AB, Luna-Villalobos Y, Velasco-Morales A, Calderón-Dávila N, et al. *Acute kidney injury in patients with severe COVID-19 in Mexico*. PLOS ONE. 2021;16(2):e0246595. https://doi.org/10.1371/journal.pone.0246595

19. Kissling S, Rotman S, Gerber C, Halfon M, Lamoth F, Comte D, Lhopitallier L, Sadallah S, et al. *Collapsing glomerulopathy in a COVID-19 patient*. Kidney Int. 2020;98(1):228–31. https://doi.org/10.1016/j.kint.2020.04.006
20. Cheng Y, Luo R, Wang K, Zhang M, Wang Z, Dong L, Li J, Yao Y, et al. *Kidney disease is associated with in-hospital death of patients with COVID-19*. Kidney Int. 2020;97(5):829–38. https://doi.org/10.1016/j.kint.2020.03.005
